# Two-dimensional QSAR-driven virtual screening for potential therapeutics against *Trypanosoma cruzi*

**DOI:** 10.3389/fchem.2025.1600945

**Published:** 2025-06-11

**Authors:** Naseer Maliyakkal, Sunil Kumar, Ratul Bhowmik, Harish Chandra Vishwakarma, Prabha Yadav, Bijo Mathew

**Affiliations:** ^1^ Department of Basic Medical Sciences, College of Applied Medical Sciences, King Khalid University, Khamis Mushait, Saudi Arabia; ^2^ Department of Pharmaceutical Chemistry, Amrita School of Pharmacy, AIMS Health Sciences Campus, Amrita Vishwa Vidyapeetham, Kochi, India; ^3^ Department of Pharmaceutical Chemistry, School of Pharmaceutical Education and Research, Jamia Hamdard, New Delhi, India

**Keywords:** Chagas disease, *Trypanosoma cruzi*, quantitative structure activity relationships, machine learning, artificial neural network, virtual screening, molecular docking, molecular dynamics

## Abstract

*Trypanosoma cruzi* is the cause of Chagas disease (CD), a major health issue that affects 6–7 million individuals globally. Once considered a local problem, migration and non-vector transmission have caused it to spread. Efforts to eliminate CD remain challenging due to insufficient awareness, inadequate diagnostic tools, and limited access to healthcare, despite its classification as a neglected tropical disease (NTD) by the WHO. One of the foremost concerns remains the development of safer and more effective anti-Chagas therapies. In our study, we developed a standardized and robust machine learning-driven QSAR (ML-QSAR) model using a dataset of 1,183 *Trypanosoma cruzi* inhibitors curated from the ChEMBL database to speed up the drug discovery process. Following the calculation of molecular descriptors and feature selection approaches, Support Vector Machine (SVM), Artificial Neural Network (ANN), and Random Forest (RF) models were developed and optimized to elucidate and predict the inhibition mechanism of novel inhibitors. The ANN-driven QSAR model utilizing CDK fingerprints exhibited the highest performance, proven by a Pearson correlation coefficient of 0.9874 for the training set and 0.6872 for the test set, demonstrating exceptional prediction accuracy. Twelve possible inhibitors with pIC_50_ ≥ 5 were further identified through screening of large chemical libraries using the ANN-QSAR model and ADMET-based filtering approaches. Molecular docking studies revealed that F6609-0134 was the best hit molecule. Finally, the stability and high binding affinity of F6609-0134 were further validated by molecular dynamics simulations and free energy analysis, bolstering its continued assessment as a possible treatment option for Chagas disease.

## 1 Introduction

Chagas disease (CD), caused by the protozoan parasite *T*rypanosoma *cruzi*, was diagnosed for the first time in humans in 1909 (Abras et al., 2022; Rassi et al., 2010; Rassi et al., 2012). CD has shifted from a regional to a global health concern, spreading beyond vector-borne transmission. *T. cruzi* infection can occur through blood transfusion, organ transplantation, congenital transmission, and laboratory accidents (Carbajal-de-la-Fuente et al., 2022). Among these, congenital transmission is particularly alarming, affecting both endemic regions (with vectors) and non-endemic areas (without vectors), making it a growing public health threat worldwide (Carbajal-de-la-Fuente et al., 2022). CD affects 6–7 million people globally, mainly in Latin America. With migration, CD has spread from rural to urban areas and beyond endemic regions, posing a growing global health challenge. In 2010, WHO classified CD as a neglected tropical disease (NTD) and later included it in the 2021–2030 roadmap for elimination. Managing NTDs remains challenging, especially in non-endemic regions, due to low awareness, limited diagnostic guidelines, and resource redirection during the COVID-19 pandemic (Fraundorfer, 2024; Kiehl et al., 2023). These factors, along with healthcare inaccessibility for vulnerable groups, threaten progress toward 2030 targets. Control efforts focus on vector control and screening, as no vaccine exists. The complex immunology and chronic nature of CD hinder vaccine development, making prevention and early detection crucial in combating the disease (Sarabi Asiabar et al., 2024). The currently approved drugs, Benznidazole and Nifurtimox, are effective but associated with severe toxicity due to their nitro groups, which generate reactive metabolic radicals (Kannigadu and N’Da, 2020; Trovato et al., 2020). This leads to adverse effects such as mutagenicity, genotoxicity, and carcinogenicity, limiting their long-term therapy (Kannigadu and N’Da, 2020). Given these challenges, the search for safer and more selective therapeutic alternatives has gained at this momentum. Since the 1990s, researchers have focused on sterol 14α-demethylase (CYP51) inhibitors, which is a crucial target in the sterol biosynthesis pathway of parasite (Choi et al., 2014; Lepesheva et al., 2011; Patterson and Fairlamb, 2019). These inhibitors offer greater selectivity and potentially reduced toxicity, making them promising candidates for improved CD treatment. However, clinical development of these compounds remains challenging, requiring further optimization to balance efficacy and safety. CYP51, a key enzyme in sterol biosynthesis, is essential for parasite survival, making it a promising drug target. Azoles like posaconazole and ravuconazole inhibit CYP51 by interacting with its heme iron, offering potential for selective treatment (Lepesheva et al., 2007; Parker et al., 2014; Rabelo et al., 2017). Other targets include cruzipain, pyrophosphate enzymes, and trypanothione reductase, though many inhibitors have shown high toxicity. Despite extensive research, benznidazole and nifurtimox remain the only FDA-approved drugs. Recent studies suggest piperazine analogues of fenarimol as safer alternatives, highlighting the need for novel and less toxic therapies (Mazzeti et al., 2021; Salomao et al., 2016; Zobi and Algul, 2025). The new derivatives with amide, sulfonamide, aromatic, carbamate, and carbonate substituents were evaluated for their ability to inhibit *T. cruzi in vitro* and showed very promising results (Keenan et al., 2013; Keenan and Chaplin, 2015).

Despite 2 decades of research, no more effective and less toxic therapeutic alternatives have been identified, and existing drug combinations remain under clinical evaluation (Cheesman et al., 2017; Harrison et al., 2020). Cheminformatics and molecular modelling offer a valuable approach, providing cost-effective solutions compared to traditional drug discovery methods (Baldi, 2010; Bayat Mokhtari et al., 2017; Siddiqui et al., 2025). QSAR is a statistical approach that correlates molecular descriptors with biological activity, aiding in the prediction of compounds with more effectiveness (Naithani and Guleria, 2024; Patel et al., 2014; Winkler, 2002). In this study, we developed a robust 2-dimensional machine learning QSAR model using a dataset of *T. cruzi inhibitors* from the ChEMBL database (https://www.ebi.ac.uk/chembl/) to predict biological activity. The model was trained on multiple molecular descriptors to establish a robust structure-activity relationship, enabling accurate activity predictions for new compounds. To further validate potential candidates, we performed virtual screening using molecular docking to assess binding affinity within the target site. The top-ranked compounds were further subjected to molecular dynamics simulations to evaluate their stability and interactions over time, ensuring their potential effectiveness as novel *T. cruzi* inhibitors.

## 2 Materials and methods

### 2.1 Data curation

To construct a machine learning-driven quantitative structural activity relationship model (ML-QSAR), we retrieved a dataset of 1,183 *T. cruzi* inhibitors along with their chemical structures as Simplified Molecular Input Line Entry System (SMILES) and biological data as maximum inhibitory concentration (IC_50_) values from the ChEMBL database (https://chembl.gitbook.io/chembl-interface-documentation/web-services). The data curation was carried out using chembl web resource client Python module. To ensure a normalized scale for the ML-QSAR model as well as to reduce variability in data analysis, the IC_50_ values were converted to pIC_50_, i.e., negative logarithm (base 10) of IC_50_.

### 2.2 Molecular descriptor calculation and feature selection

We used padelpy (https://github.com/ecrl/padelpy), a Python wrapper of the PaDEL-descriptor software, to calculate 1,024 CDK fingerprints and 780 atom pair 2D fingerprints (Yap, 2011) for the retrieved 1,183 inhibitors. Following the descriptor calculation step, we further implemented variance threshold scores and Pearson correlation analysis-based selection (correlation coefficient >0.9) to eliminate the constant and highly correlated features, respectively, from both the fingerprint datasets.

### 2.3 ML-QSAR model development and evaluation

We used an 80:20 split ratio for generating the training and test datasets for both fingerprints. We implemented Support Vector Machine (SVM), Artificial Neural Network (ANN), and Random Forest (RF) ML algorithms to develop individual QSAR models for each of the fingerprint datasets using the scikit-learn (https://scikit-learn.org/stable/) Python programming library (Breiman, 2001; Goel et al., 2023; Utkin, 2019). For the SVM model, we implemented the radial basis function (RBF) kernel to capture the non-linear relationships between the molecular fingerprints and biological activity. Additionally, the model was optimized for C (regularization) and gamma (kernel coefficient) parameters. In case of the ANN-driven QSAR model, we implemented a feedforward neural network (FNN) with one hidden layer. We also tuned the number of neurons, activation function (ReLU), and optimizer (Adam) for the ANN model. For the RF-driven QSAR model, an ensemble of decision trees was used along with a feature bagging technique to enhance the predictive power of the model. Additionally, we also optimized the number of estimators (trees), the depth of trees, and the minimum samples per split.

Following the development of the initial model, we further performed principal component analysis (PCA) to assess the distribution of compounds and detect potential outliers in both training and test datasets. PCA was applied to transform the high-dimensional descriptor space into principal components, retaining maximum variance in a low-dimensional space. The first two principal components were further visualized using a scatter plot to inspect cluster formation as well as to detect points deviating from the main distribution. Molecules falling outside the main data clusters were detected as outliers and were removed from further modeling.

Following outlier detection and removal, we further trained the model using grid-based hypertuning and cross-validation metrics using SVM, ANN, and RF algorithms. To determine the best-performing models, we computed a diverse set of statistical metrics for each model: root mean squared error (RMSE), mean squared error (MSE), mean absolute error (MAE), Pearson Correlation coefficient, and 10-fold cross-validation metrics. The model with the lowest RMSE, MSE, and MAE values while retaining a high Pearson Correlation Coefficient was selected as the optimal QSAR model for predicting the inhibition mechanism.

### 2.4 Feature elucidation of the ML-QSAR model for rational drug-design

To further enhance the interpretability of the ML-QSAR models for both the fingerprints, we further implemented different feature importance analysis techniques like Variance Importance in Projection Analysis (VIP), Correlation Matrix Analysis, and Shapley Additive Explanations (SHAP) (https://shap.readthedocs.io/en/latest/) analysis (Nohara et al., 2022). For the VIP plot analysis, we computed the Partial Least Squares Regression (PLSR) method to rank descriptors based on their contribution to the model (Cao et al., 2017). For Correlation Matrix analysis, a pairwise correlation matrix was generated to identify the positively and negatively correlated molecular fingerprints for both models. For SHAP analysis, we individually computed SHAP values for each fingerprint to interpret how each molecular fingerprint influenced the pIC_50_ values across the datasets. Additionally, we also did cluster analysis for both highly active (pIC_50_≥7) and weak inactive molecules (pIC_50_≤4) using Tanimoto Coefficient-based similarity analysis (https://github.com/MunibaFaiza/tanimoto_similarities). This clustering approach helped in identifying shared molecular features among compounds demonstrating strong or weak inhibition activity. To further enhance the interpretability of the clustering approach, we employed a WordCloud (https://pypi.org/project/wordcloud/) approach to visualize the most frequently occurring molecular features among the two groups.

### 2.5 Machine learning-driven chemical library screening

To further pave the path for the discovery of novel and more effective drug candidates, we implemented a two-dimensional multiplex modeling to screen large chemical libraries. We curated an Antiprotozoal Screening Compound Library of 8,200 molecules from the Life Chemicals database (https://lifechemicals.com/screening-libraries/targeted-and-focused-screening-libraries/antiprotozoal-library). We then implemented the first layer of virtual screening approach, i.e., pharmacokinetic and toxicophore analysis of the molecule library through the ChemBioServer 2.0 (https://chembioserver.vi-seem.eu/). We initially screened the molecules through Lipinski’s Rule of Five, Veber’s Rule, and Ghose’s Filter using the ChemBioServer 2.0. Following this, we again used the ChemBioServer 2.0 to utilize the toxiphore analysis approach to screen out the toxic molecules (Karatzas et al., 2020). The screened molecules were then subjected to the second layer of virtual screening, i.e., activity prediction and screening using our previously developed ML-QSAR for both the molecular feature datasets, i.e., CDK and atom 2D pair fingerprints.

### 2.6 Molecular docking

On the aforementioned conformations, molecular docking studies were performed to investigate residue interactions and binding energy scores of lead molecules from chemical library screening. For the current investigation, the drug target, cruzain enzyme from *T. cruzi* (PDB ID: 1ME3) (Huang et al., 2003) was retrieved from the protein data bank (https://www.rcsb.org/) with a superior resolution of 1.2 Å, bearing a co-crystallized ligand*.* Missing residues were restored with the glide after the existing ligands were removed and hydrogen atoms were added. The co-crystallized ligand has been redocked to the active site of the 1ME3 to ascertain the docking parameters. Molecular docking employed a three-step approach that comprised protein energy reduction using the Protein Preparation Wizard (PPW) tool, optimization, and pre-processing to create protein crystal structures. LigPrep was utilized to create the ligands, guaranteeing accurate assignment of atom types and protonation states at pH 7.4 ± 1.0. Hydrogen atoms were added, and the structures underwent bond ordering. Then, using the Receptor grid generating tool (Kumar et al., 2024; Rampogu et al., 2018), a grid was created at the binding pocket coordinates (x, y, z) and aligned with a co-crystallized ligand.

### 2.7 Molecular dynamics simulation (MDS)

The “Desmond V 7.2 package” (Schrodinger 2022-4) has been used to conduct MDS to examine how the solvent system affects the structure of the protein-ligand complex. The simulations were done on a Dell Inc. Precision 7,820 Tower running Ubuntu 22.04.1 LTS 64-bit and outfitted with an Intel Xeon (R) Silver 4210R processor and an NVIDIA Corporation GP104GL (RTX A 4000) graphics processing unit. The docked complex’s MDS (F6609-0134-1ME3) was performed using the OPLS4 force field. For MDS, the complex is positioned in the middle of an orthorhombic cubic box. After adding SPC water molecules and buffers, the protein atom and the edge of the box are separated by 10 Å using the NPT ensemble. Together with counterions like Na+ and Cl-injected to randomly neutralize the system, the boundary condition box volume has also been calculated depending on the complex type. To assess domain correlations, a study of the protein-ligand interaction, root mean square deviation (RMSD), and root mean square fluctuation (RMSF) was conducted over all Cα atoms during the 200 ns MD simulation (da Costa et al., 2022; Maliyakkal et al., 2024).

## 3 Results and discussion

### 3.1 ML-QSAR model development

Following the removal of constant features and highly correlated features using variance threshold and correlation-based feature elimination approaches, we retained 533 molecular features for the CDK fingerprint Dataset, and 25 molecular features for the atom pair 2D fingerprint dataset. Following this, we followed an 80:20 split for both fingerprint datasets before model development, individually for the two fingerprint datasets. The split resulted in 946 molecules in the training set and 237 molecules in the test set. We further implemented SVM, ANN, and RF-based ML algorithms using the generated training and test datasets to develop individual ML-QSARs for two different molecular features, i.e., CDK and atom 2D pair fingerprint datasets.

**FIGURE 1:**
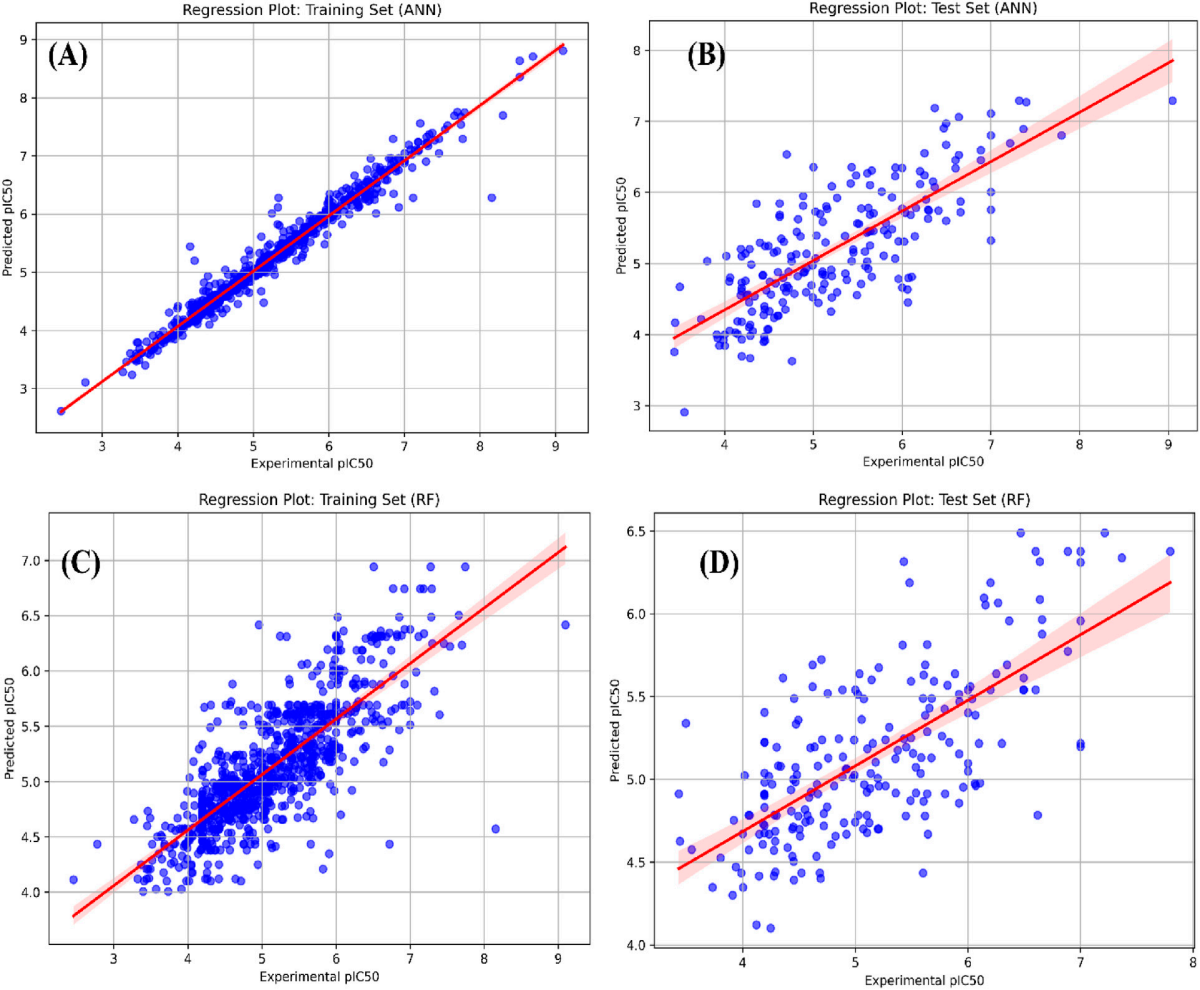
**(A,B)** represent regression plots for the training and test datasets for the best CDK fingerprint-driven ANN-QSAR model, respectively; **(C,D)** represent regression plots for the training and test datasets for the best atom 2D pair fingerprint-driven RF-QSAR model.

For the CDK fingerprint model, the ANN-driven QSAR model was found to show the best statistics and model accuracy for the initial run. The model demonstrated a Pearson correlation coefficient of 0.9874 and 0.6872, RMSE of 0.1511 and 0.7271, MSE of 0.0228 and 0.5287, and MAE of 0.0.086 and 0.5614, for training and test datasets, respectively. Following the initial model run, we further implemented PCA analysis to detect the outliers in the datasets. The PCA-based clustering revealed that 119 molecules from both the training and test datasets deviated significantly from the main cluster and were classified as outliers. These molecules were removed from both the training and test datasets before final model deployment. Following outlier removal, we optimized hyperparameters for all 3 ML algorithms using randomized search cross-validation. For the RF model, we set the n_estimators between 100 and 700, the max_depth range between 10 and 30, and the min_sample_split range between 2 and 6. For the SVM model, we optimized the C value between 1 and 100, the gamma value range between 0.01 and 0.0001, the epsilon value between 0.01 and 0.2, and kernel type as “rbf,” “poly,” “sigmoid.” Lastly, for the RF model, we optimized hidden layer size to [(128,64), (256,128,64), (512,256,128)], set activation functions to “relu” and “tanh”, and solver to “adam” and “sdg.” For the RF model, the learning rates and maximum iterations were kept in the range of 0.001–0.1 and 1,000–2,000, respectively. Even after hyperparameter optimization, the ANN model demonstrated the best performance for the CDK fingerprints by demonstrating a Pearson correlation coefficient of 0.9845 and 0.7683, RMSE of 0.1674 and 0.6167, MSE of 0.028 and 0.3804, and MAE of 0.0915 and 0.4842, for the training and test datasets, respectively. Additionally, it demonstrated a 10-fold cross-validation of 0.7870. Overall, it was evident that the hypertuned CDK fingerprint-driven ANN-QSAR shows the best accuracy and robustness among all the trained models (Figure 1; Table 1).

**TABLE 1:** Statistical metrics of all the generated CDK fingerprint-driven QSAR models. Best model is in bold.

Algorithm	Train RMSE	Test RMSE	CV RMSE	Train MSE	Test MSE	Train MAE	Test MAE	Train pearson	Test pearson
Initial models									
RF	0.2659	0.5986	0.6908	0.0707	0.3583	0.1943	0.4607	0.9656	0.7630
SVM	0.4449	0.5887	0.6682	0.1980	0.3465	0.2755	0.4493	0.8845	0.7735
ANN	0.1512	0.7272	0.8266	0.0229	0.5288	0.0868	0.5614	0.9875	0.6872
Final models (hypertuned and outliers removed)									
RF	0.3413	0.5692	0.6824	0.1165	0.3240	0.2518	0.4417	0.9441	0.7985
SVM	0.4827	0.6345	0.7237	0.2330	0.4026	0.3029	0.4835	0.8641	0.7415
ANN (best model)	**0.1675**	**0.6168**	**0.7870**	**0.0280**	**0.3804**	**0.0915**	**0.4842**	**0.9846**	**0.7683**

For the atom 2D fingerprint model, the RF-driven QSAR model was found to show the best statistics and model accuracy for the initial run. The model demonstrated a Pearson correlation coefficient of 0.86088 and 0.73814, RMSE of 0.47631 and 0.62489, MSE of 0.22687 and 0.39084, and MAE of 0.33354 and 0.49899, for the training and test datasets, respectively. Additionally, the 10-fold cross-validation for the initial run of the RF-QSAR model was performed. Following the initial model run, we further implemented PCA analysis to detect the outliers in the datasets. The PCA-based clustering revealed that 118 molecules from both the training and test datasets deviated significantly from the main cluster and were classified as outliers. These compounds were removed from both the training and test datasets before final model deployment. Following outlier removal, we optimized hyperparameters for all 3 ML algorithms using randomized search cross-validation. We kept the same hyperparameters for the 3 ML algorithms that we implemented previously for the CDK fingerprint-based QSAR models. Even after hyperparameter optimization, the RF model demonstrated the best performance for atom 2D pair fingerprints by demonstrating a Pearson correlation coefficient of 0.79293 and 0.69248, RMSE of 0.5438 and 0.63825, MSE of 0.2957 and 0.4073, and MAE of 0.4111 and 0.5184, for training and test datasets, respectively. Additionally, it demonstrated a 10-fold cross-validation of 0.7161. Since there was a reduction in the statistical robustness of the outlier-driven hypertuned model, we implemented the same hyperparameter optimization on the original dataset without outlier removal. We observed that even though the RF algorithm performed better than the outlier-driven hypertuned RF model, it still demonstrated low accuracy and robustness as compared to the initial RF-QSAR model with default parameters. Thereby, we concluded that the initial RF-QSAR model without outlier analysis demonstrated the best performance for the atom 2D pair fingerprint dataset (Figure 1; Table 2).

**TABLE 2:** Statistical metrics of all the generated atom 2D pair fingerprint-driven QSAR models. Best model is bold.

Algorithm	Train RMSE	Test RMSE	CV RMSE	Train MSE	Test MSE	Train MAE	Test MAE	Train pearson	Test pearson
Model (default parameters)									
RF (best model)	**0.4763**	**0.6249**	**0.7472**	**0.2269**	**0.3905**	**0.3335**	**0.4990**	**0.8609**	**0.7381**
SVM	0.6141	0.6925	0.7331	0.3771	0.4795	0.4222	0.5514	0.7549	0.6655
ANN	0.4851	0.7449	0.8031	0.2354	0.5548	0.3392	0.5731	0.8595	0.6319
Model (hypertuned)									
RF	0.4846	0.6237	0.7415	0.2348	0.3891	0.3446	0.5007	0.8561	0.7397
SVM	0.7839	0.8239	0.8147	0.6145	0.6788	0.5964	0.6568	0.5404	0.4691
ANN	0.4639	0.6992	0.8298	0.2152	0.4888	0.3047	0.5444	0.8671	0.6698
Model (hypertuned and outliers removed)									
RF	0.5438	0.6383	0.7162	0.2958	0.4074	0.4112	0.5185	0.7929	0.6925
SVM	0.6314	0.6979	0.7286	0.3987	0.4870	0.4554	0.5593	0.6877	0.6008
ANN	0.5910	0.7095	0.7697	0.3493	0.5034	0.4489	0.5630	0.7479	0.6044

### 3.2 Feature elucidation of the ML-QSAR model for rational drug-design

**FIGURE 2:**
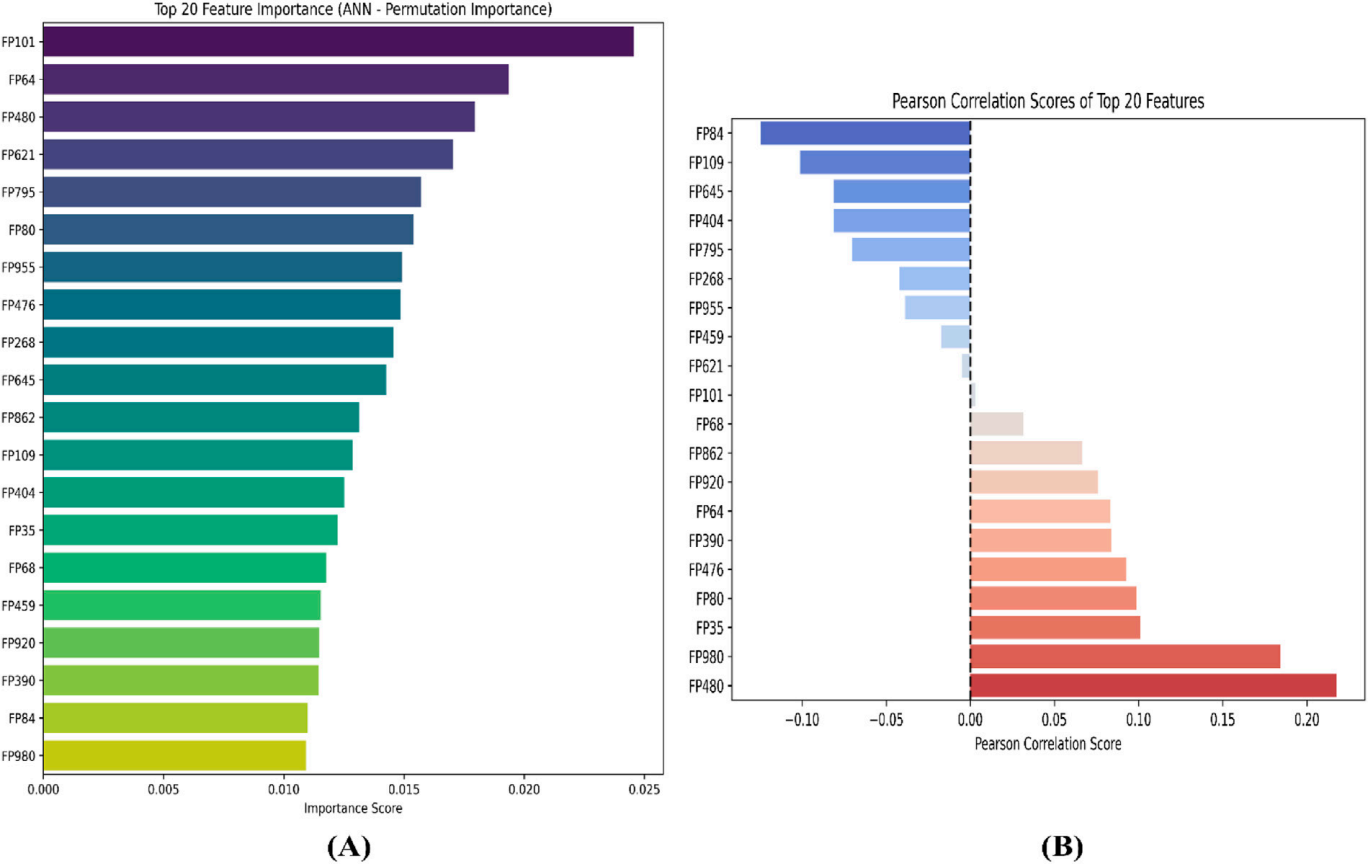
Representation of top 20 features from VIP plot **(A)** and Pearson correlation plot **(B)** for the CDK fingerprint-driven ANN-QSAR model.

**FIGURE 3:**
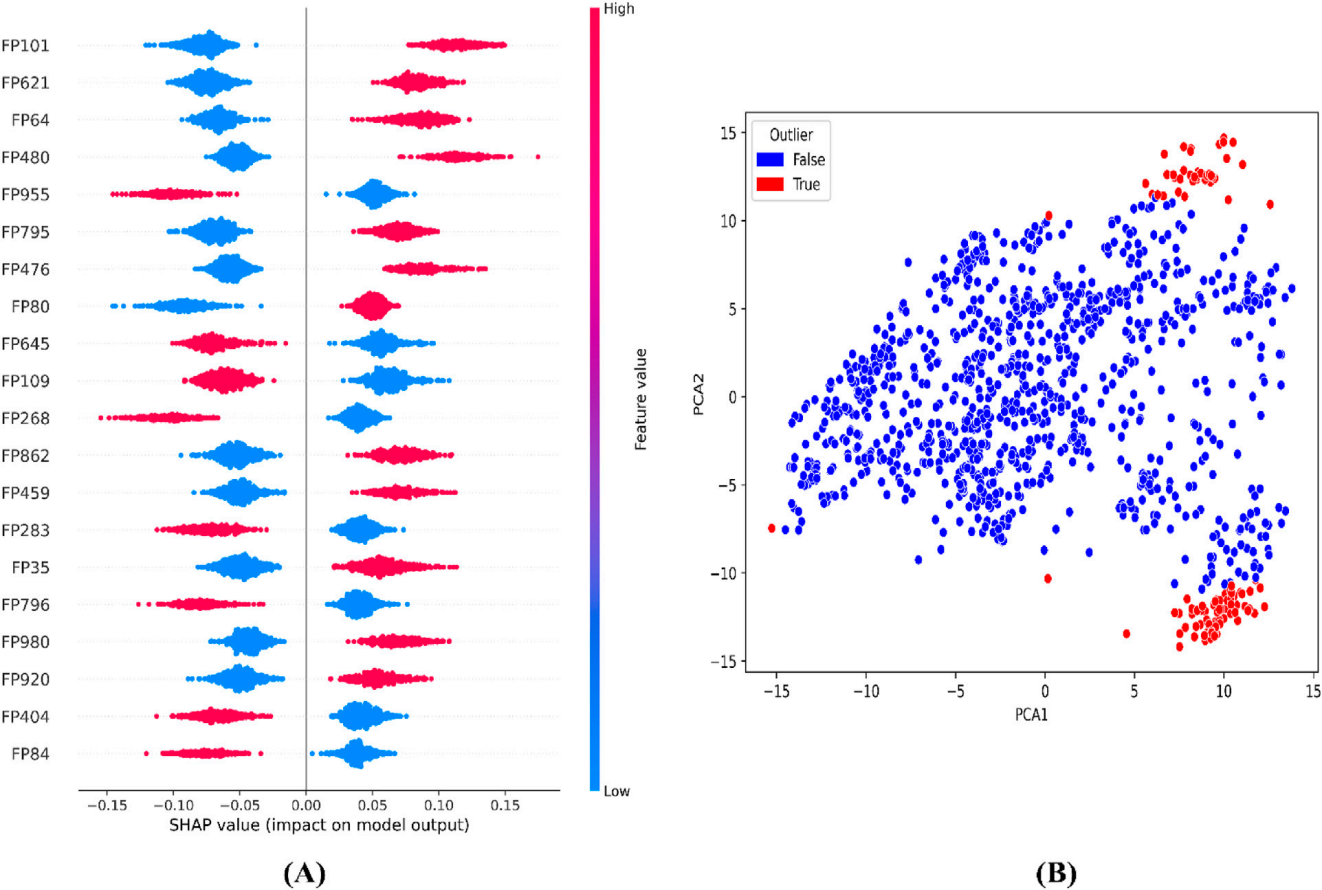
Representation of top features from SHAP analysis **(A)** and outlier analysis through PCA plot **(B)** for the CDK fingerprint-driven ANN-QSAR model.

To further interpret the top 20 significant features ML-QSAR model for rational design of novel and more efficient inhibitors, we implemented VIP plot, correlation matrix, and SHAP analysis. For the CDK fingerprint-driven ANN QSAR, we observed that fingerprints, like FP101, FP64, FP480, FP980, and FP35, demonstrated high variance threshold scores in VIP plot analysis, suggesting that the presence of these features in the inhibitor molecule might lead to an increase in biological activity (pIC_50_ value). We also observed that fingerprints, FP84, FP109, and FP645, showed low variance threshold scores in the VIP plot, thereby suggesting they might have a negative relationship with biological activity. However, to build a suggestive narrative as to whether these fingerprints in the VIP plot are positively or negatively correlated, we further investigated them through a Pearson correlation matrix and SHAP analysis. Through Pearson correlation matrix analysis, we observed that FP480, FP980, and FP35 demonstrated positive correlation scores towards biological activity, whereas FP84, FP109, and FP645 demonstrated negative correlation scores towards biological activity. Furthermore, from SHAP analysis, it was evident that fingerprints FP480, FP980, and FP64 demonstrated high feature value, thereby suggesting that their presence would lead to an increased pIC_50_ value, whereas on the other hand fingerprints FP84, FP109, and FP645, demonstrated negative SHAP values, suggesting the fact that their presence would lead to a decrease in pIC_50_ value. Additionally, we did a molecular feature-driven Tanimoto clustering analysis of the high-activity and low-activity molecules of the QSAR dataset. The cluster analysis of the high activity molecules further validated the presence of fingerprints such as FP480, FP64, FP980, and FP35, which were already visualized by Pearson correlation and SHAP analysis plots as positively correlated features. Furthermore, the cluster analysis of the low activity molecules of the QSAR dataset further validated the presence of fingerprints such as FP84, FP109, and FP645, which were already labelled as negatively correlated by the Pearson correlation matrix and SHAP analysis (Figures 2, 3, 6).

**FIGURE 4:**
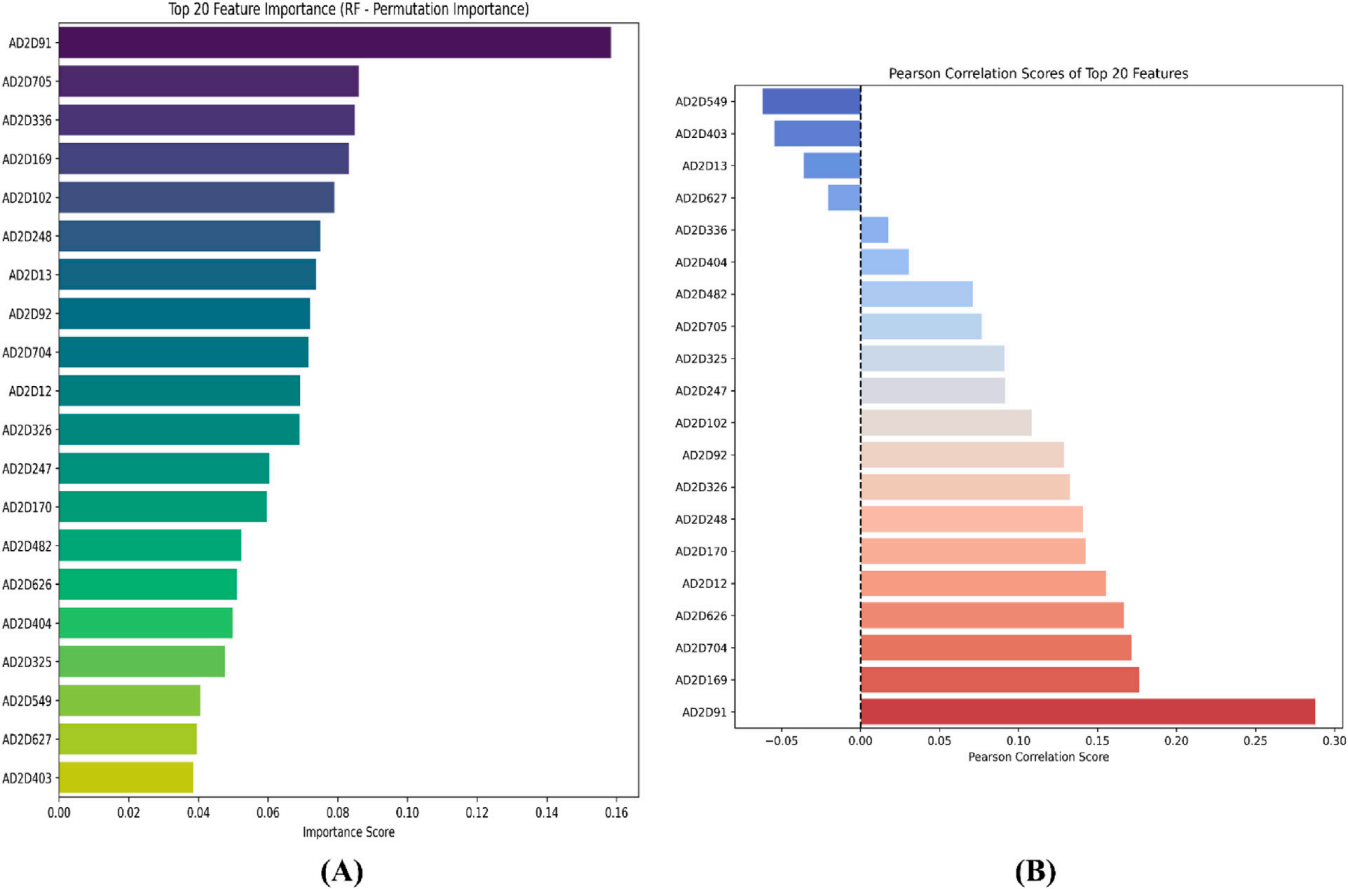
Representation of the top 20 features from VIP plot **(A)** and Pearson correlation plot **(B)** for the atom 2D pair fingerprint-driven RF-QSAR model.

**FIGURE 5:**
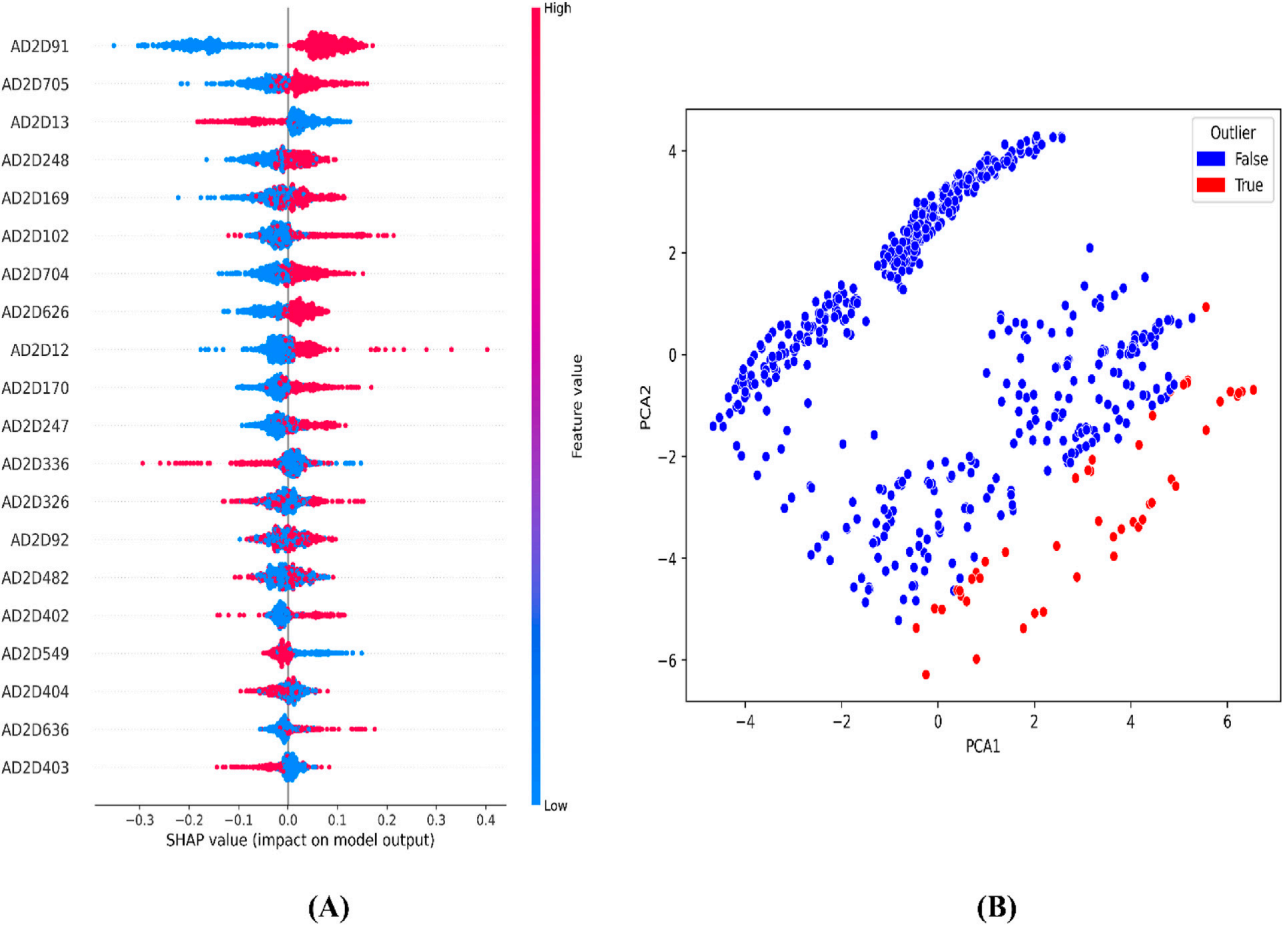
Representation of top features from SHAP analysis **(A)** and outlier analysis through PCA plot **(B)** for the atom 2D pair fingerprint-driven RF-QSAR model.

**FIGURE 6:**
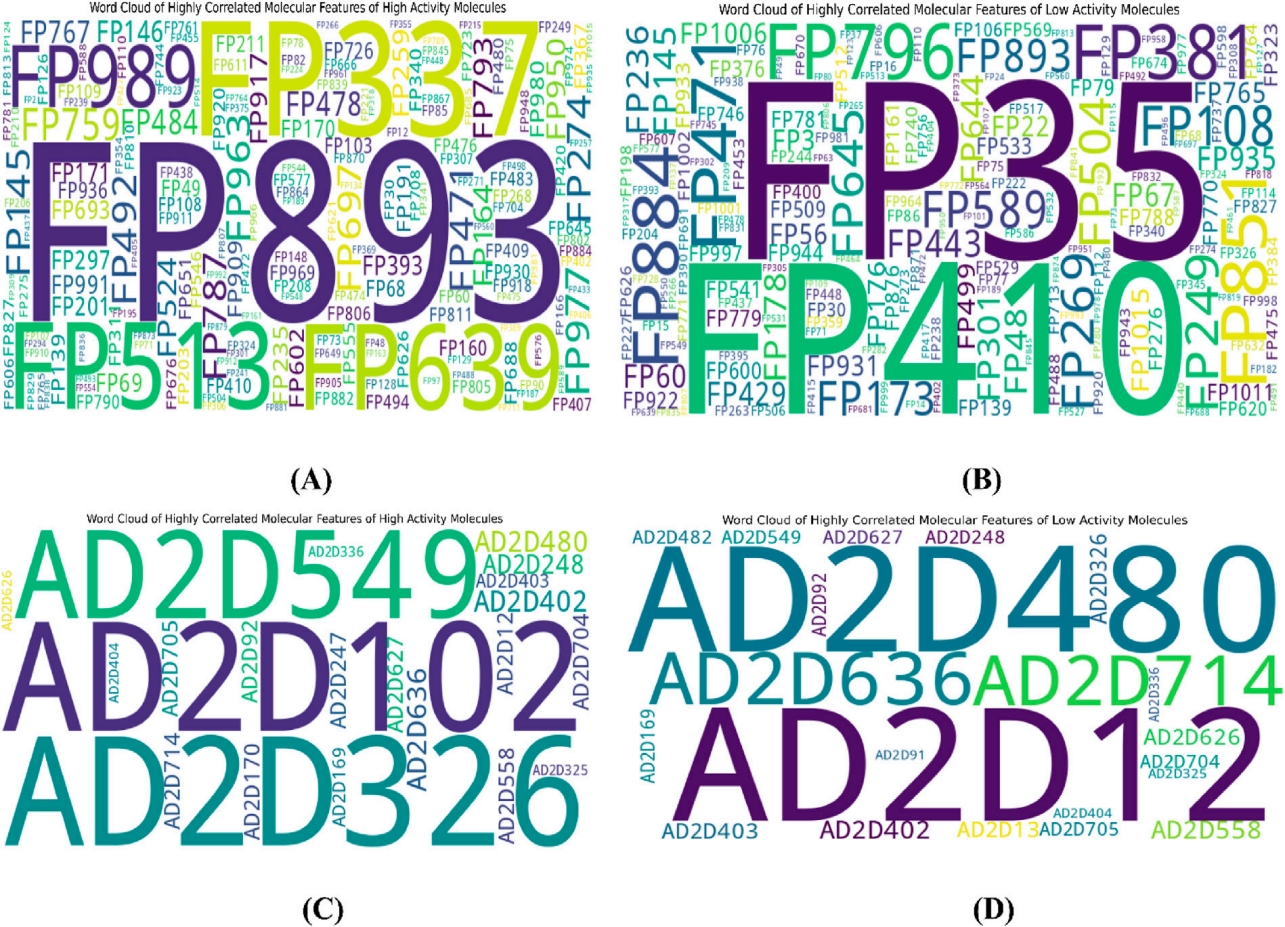
Representation of top features through Tanimoto similarity-driven cluster analysis of highly active **(A)** and low active molecules **(B)** from the CDK fingerprint dataset; highly active **(C)** and low active **(D)** molecules from the atom 2D pair fingerprint dataset.

For the atom 2D pair fingerprint-driven RF-QSAR model, it was evident that features AD2D91 (presence of N-N at topological distance 2), AD2D705 (presence of C-O at topological distance 10), AD2D336 (presence of O-O at topological distance 5), AD2D169 (presence of N-N at topological distance 3), AD2D102 (presence of O-O at topological distance 2), AD2D248 (presence of N-O at topological distance 4), and AD2D13 (presence of N-N at topological distance 1), demonstrated high variance threshold scores through the VIP plot analysis. We also observed that molecular features like, AD2D92 (presence of N-O at topological distance 2), AD2D704 (presence of C-N at topological distance 10), AD2D12 (presence of C-X at topological distance 1), AD2D247 (presence of N-N at topological distance 4), AD2D326 (presence of N-O at topological distance 5), AD2D170 (presence of N-O at topological distance 3), AD2D482 (presence of N-O at topological distance 7), AD2D626 (presence of C-N at topological distance 9), AD2D404 (presence of N-O at topological distance 6), AD2D325 (presence of N-N at topological distance 5), AD2D549 (presence of C-O at topological distance 8), AD2D627 (presence of C-O at topological distance 9), and AD2D403 (presence of N-N at topological distance 6) demonstrated moderate to low variance threshold score through VIP plot analysis. To further investigate the nature of the correlation of the VIP plot-derived features, we conducted a Pearson correlation matrix and SHAP analysis. Through the Pearson correlation matrix analysis, it was evident that fingerprints AD2D91, AD2D169, AD2D704, AD2D626, and AD2D12 demonstrated positive correlation with high biological activity, whereas fingerprints AD2D170, AD2D248, AD2D326, AD2D92, AD2D102, AD2D325, and AD2D705 demonstrated moderately positive correlation with biological activity. Additionally, the Pearson correlation matrix also demonstrated that fingerprints, AD2D549, AD2D403, AD2D13, AD2D627, AD2D336, AD2D404, and AD2D482 showed negative correlation with biological activity. For SHAP analysis, it was observed that AD2D91, AD2D169, AD2D170, and AD2D12 demonstrated higher SHAP values, suggesting their positive impact on biological activity, whereas AD2D13, AD2D248, AD2D336, and AD2D705 showcased negative SHAP values, thereby demonstrating their negative impact on biological activity. We also did a molecular feature-driven Tanimoto clustering analysis of the high-activity and low-activity molecules of the QSAR dataset. We observed that positively correlated features such as AD2D549, AD2D102, AD2D336, AD2D92, AD2D248, AD2D404, AD2D704, AD2D170, AD2D403, AD2D626, AD2D12, and AD2D326 from the Pearson correlation matrix and SHAP analysis were also found to be present in the cluster analysis of highly active molecules of the dataset. On the other hand, molecular features like AD2D480, which were labelled to be negatively correlated to biological activity in both SHAP and Pearson correlation matrix analysis, were found to be significantly present in cluster analysis of low activity molecules of the QSAR dataset (Figures 4–6).

### 3.3 Machine learning-driven chemical library screening

We initially screened the Antiprotozoal Screening Compound Library of 8,200 molecules from the Life Chemicals database using the combination of Lipinski’s Rule of Five, Veber’s Rule, and Ghose’s Filter through the ChemBioServer 2.0. A total of 133 molecules that passed through this filtration step were further subjected to toxiphore analysis. A total of 93 out of 133 molecules were found to pass the toxicophore analysis study. Following this, we further predicted the biological activity (pIC_50_ value) of the 93 molecules using our previously developed CDK fingerprint-driven RF-QSAR and atom 2 days pair fingerprint-driven ANN-QSAR model. We then calculated the cumulative of the predicted biological activities for each molecule from both models to identify the most promising inhibitor. To streamline the drug discovery process further, we identified 12 molecules with pIC_50_ ≥ 5 (Table 3).

**TABLE 3:** Top 12 hit molecules bioactivity prediction scores using our previously developed ML-QSAR models.

Name	Predicted_pIC_50__a2d_fp	Predicted_pIC_50__cdk_fp	Predicted_pIC_50__cumulative
F2207-0115	4.9917	6.9805	5.9861
F2207-0102	5.2784	6.6665	5.9725
F6548-1609	5.1616	6.6195	5.8906
F6548-3996	5.3880	6.1246	5.7563
F6609-0134	5.9258	5.5255	5.7256
F6619-3684	5.9071	5.4719	5.6895
F2014-0155	5.5000	5.8509	5.6754
F6609-0164	5.5789	5.7068	5.6428
F3222-1452	4.9646	6.2789	5.6217
F0507-2033	6.8440	4.313	5.5785
F1872-0526	4.5384	6.6049	5.5716
F0676-0414	6.8440	4.2031	5.5236

### 3.4 Molecular docking

**FIGURE 7:**
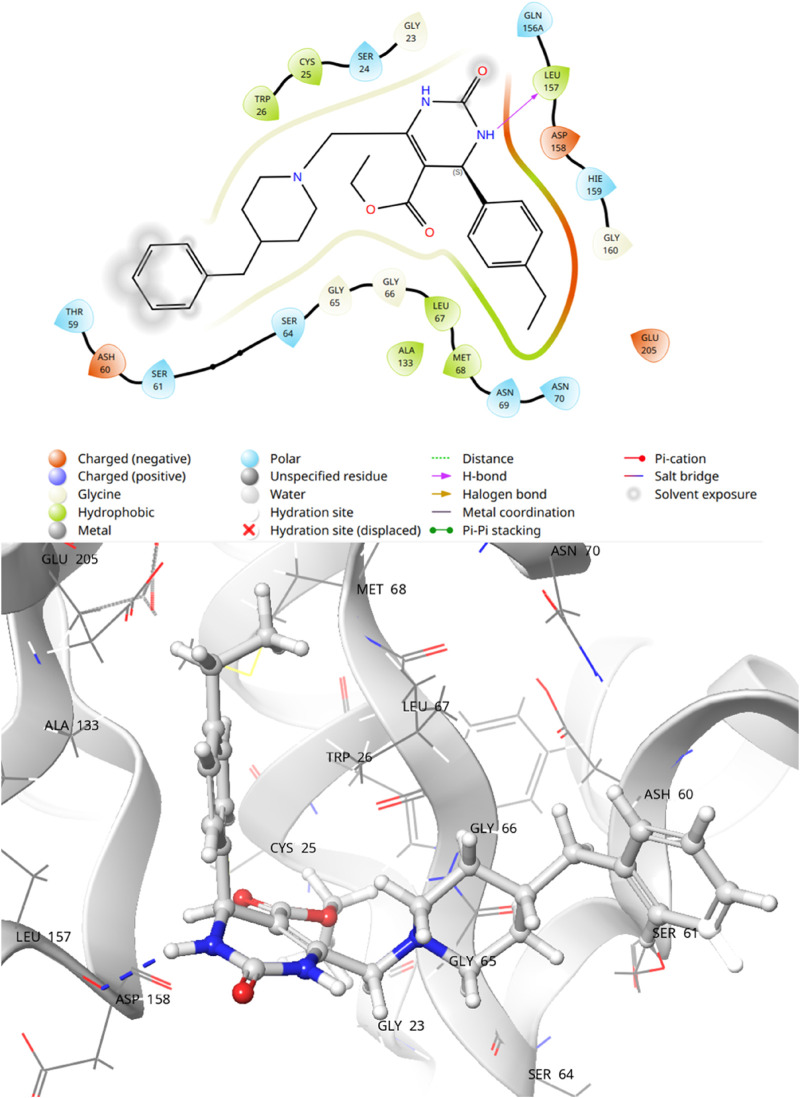
2-D and 3D interaction of Lead compound F6609-0134 with binding pocket of 1ME3.

Molecular docking studies were conducted to have a better understanding of the lead compound’s binding processes. Lead compounds found by virtual screening coupled with the 1ME3 protein, and the docking procedure was confirmed using native ligands. We also did a comparative docking analysis with the native ligand P10 (PubChem CID: 5289091). P10 molecule has already been experimentally tested as an active against *T. cruzi* (BioAssay AID: 977610; BioAssay AID: 1811). In the previous study on the 3D crystal structure of 1ME3, inhibitors were found to form a strong hydrogen bond with His159, part of the canonical catalytic triad (Cys25, His159, Asn175). The P2-position phenylalanine fits into the hydrophobic S2 pocket formed by Leu67, Ala133, and Leu157, with Glu205 rotating to accommodate the side chain. The inhibitor backbone is stabilized by hydrogen bonds with Gly66 and Asp158, along with key water-mediated interactions. Additionally, the nitrogen atom of inhibitors shows potential interaction with the hydroxyl group of Ser61. The compounds mentioned in Table 4 have docking scores (XP mode) for 1ME3 ranging from −3.843 to −6.352 kcal/mol. With a docking score of −6.352 kcal/mol, F6609-0134 had the highest binding affinity of all of them, whereas the co-ligand received a value of −6.023 kcal/mol (Table 4). F6609-0134 established a hydrogen connection with Leu157, more precisely with the NH atom of the pyrimidine ring, according to an analysis of the 2-D and 3-D interaction map. Hydrophobic interactions were also noted with Asp158, Gly160, Glu205, Leu67, Met68, Cys25, Trp26, Thr59, Ser61, and Ser64 (Figure 7). Furthermore, the lead chemical demonstrated hydrogen bonding with significant residues, as previously reported in the literature and discussed above regarding the binding pocket (Durrant et al., 2010; Rampogu et al., 2018; Rogers et al., 2012; Wiggers et al., 2013; Huang et al., 2003). The discovered hits may be promising lead candidates for the therapy of Chagas disease, as the lead compound had lower binding energies and higher docking scores than the reference compounds.

**TABLE 4:** Docking score of 1ME3 with lead molecules from virtual screening, and native ligand. Best docking score is in bold.

Compound	Docking score (kcal/mol)	Compound	Docking score (kcal/mol)
F2207-0115	−5.052	F6609-0164	−4.842
F2207-0102	−4.368	F3222-1452	−5.252
F6548-1609	−3.843	F0507-2033	−5.389
F6548-3996	−4.236	F1872-0526	−4.864
**F6609-0134**	**−6.352**	F0676-0414	−5.479
F6619-3684	−3.868	Co-ligand	−6.023
F2014-0155	−4.94		

### 3.5 Molecular dynamics

**FIGURE 8:**
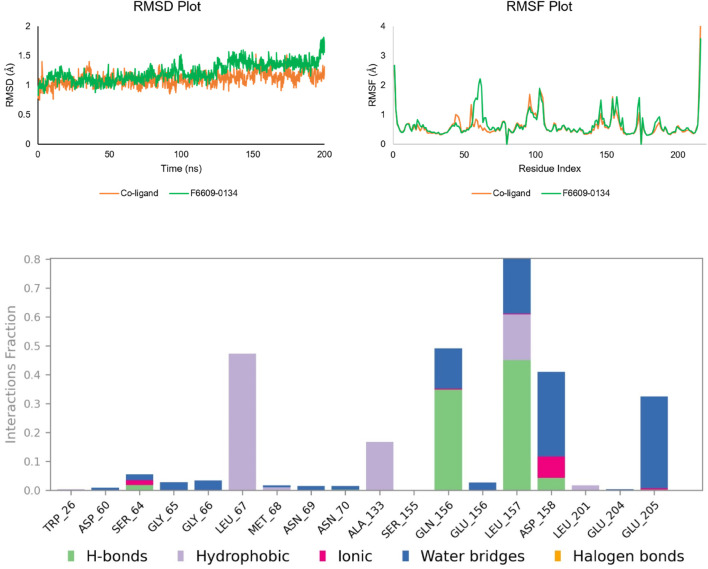
Analysis of the inhibitor-ligand complex using MD simulation: RMSD plot (co-crystallized ligand RMSD is shown in orange, and RMSD of F6609-0134 is shown in green); RMSF plot (co-crystallized ligand RMSF is shown in orange, and RMSF of F6609-0134 is shown in green); and analysis of protein-ligand contacts of the MD trajectory of the F6609-0134-1ME3 complex.

To investigate the flexibility and stability of the docked complex of F6609-0134 at the binding site of the 1ME3 protein in biological situations, MD simulations were performed. We also performed a comparative MD analysis of our hit molecule against P10 molecules (co-crystallized native ligand). MD trajectories were used to calculate protein-ligand interactions as well as RMSD and RMSF. Figure 8 shows a number of analyses of the MD trajectory data for the F6609-0134-1ME3 complex.

#### 3.5.1 Root mean square deviation

According to RMSD Figure 8, the Cα atoms of the protein in connection with F6609-0134 and co-ligand had RMSD values ranging from 0.83 to 1.80 Å and 0.74 to 1.52 Å, respectively. This suggests that the ligand-protein complex remained stable throughout the simulation. Except for a slight variation observed between 120 and 130 ns, the protein’s RMSD remained constant throughout the simulation, and the co-ligands did not differ all that much. Based on the complex’s predicted trajectory, the RMSD values of its Cα atoms demonstrated the stability of the protein-ligand complex in a dynamic environment. A higher RMSD value indicates unfolding for protein Cα atoms, whereas a smaller value indicates compactness. The modest change in the backbone RMSD further supported the equilibration of the protein-ligand combination. The difference between the highest and lowest RMSD values represented the backbone deviation. In summary, the total RMSD of the F6609-0134-1ME3 complex remains consistent and dependable in a fluctuating environment.

#### 3.5.2 Root mean square fluctuation

The flexibility of the protein system was measured during the simulation using the RMSF of each amino acid residue. The RMSF plot showed that differences in N-terminal residues were more noticeable. During the simulation, it was discovered that the co-ligand and compound F6609-0134 interacted with amino acids 19 and 18, respectively, of 1ME3. With a few exceptions (Figure 8; Table 5), all of these interacting residues had RMSF values smaller than 1 Å. Certain amino acid residues in the protein-ligand complex are essential for the stability of dynamic processes. The RMSF parameter, which is derived from the MD simulation trajectories, measures the deviation of individual amino acids from the reference or native structure. The RMSF visualization facilitates comprehension of the remaining vibrations in the F6609-0134-1ME3 complex. This finding suggests a solid binding of the lead medication with minor conformational changes within the binding pocket of the target protein, since the main chain and active site residues only slightly varied.

**TABLE 5:** Amino acid contacts with the ligand and their RMSF value.

Compound	Amino acids that come into contact with ligands and their RMSF (Å)
F6609-0134	Trp26 (0.44 Å), Asp60 (2.02 Å), Ser64 (0.89 Å), Gly65 (0.89 Å), Gly66 (0.63 Å), Leu67 (0.57 Å), Met68 (0.48 Å), Asn69 (0.50 Å), Asn70 (0.57 Å), Ala133 (0.43 Å), Ser155 (1.61 Å), Glu156 (1.18 Å), Gln156 (0.76 Å), Leu157 (0.62 Å), Asp158 (0.9 Å), Leu201 (0.48 Å), Glu204 (0.56 Å), and Glu205 (0.48 Å)
Co-ligand	Gln19 (0.46 Å), Cys25 (0.37 Å), Trp26 (0.39 Å), Thr59 (0.81 Å), Asp60 (0.55 Å), Ser61 (0.65 Å), Cys63 (0.47 Å), Ser64 (0.54 Å), Gly65 (0.48 Å), Gly66 (0.42 Å), Leu67 (0.42 Å), Met68 (0.38 Å), Asn70 (0.44 Å), Ala133 (0.39 Å), Ala136 (0.46 Å), Leu157 (0.49 Å), Asp158 (0.55 Å), His159 (0.36 Å), and Trp177 (0.49 Å)

#### 3.5.3 Protein ligand contact analysis

**FIGURE 9:**
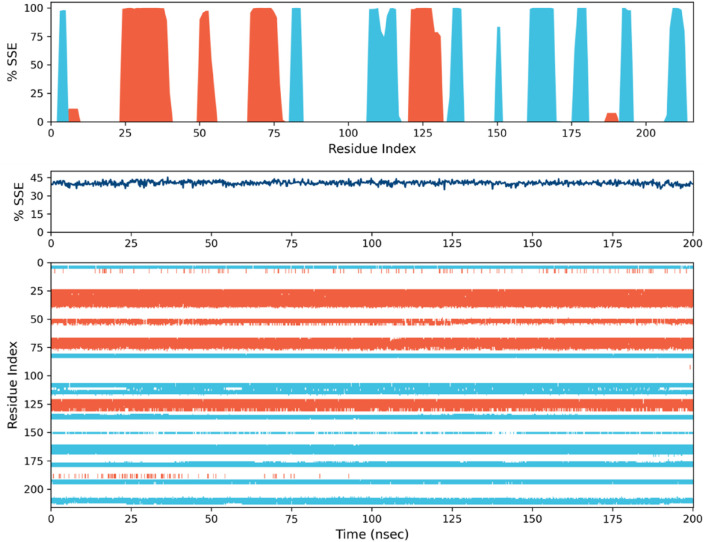
Secondary structure element (SSE) distribution plotted against residue index for 1ME3. The SSE composition across each trajectory frame throughout the simulation for 1ME3.

The most prevalent contact types, as determined by MD simulations, were hydrophobic, hydrogen bonding, and polar (water-mediated hydrogen bonding). According to a protein-ligand contact research, Leu67, Ala133, Glu156, Leu157, Asp158, and Glu205 strongly contacted F6609-0134. The simulation results show that compound F6609-0134, Leu167, Gln156, Leu157, Asp157, and Glu205 can stabilize 1ME3 protein because the particular contact is sustained for over 40% of the simulation time (Figure 8). Comparing the ligand’s 2-D interaction during docking (Figure 9) with the subsequent simulation reveals similar interactions. The Figure 7 simulation result for compound F6609-0134 indicates that it forms a hydrogen bond with amino acid Leu157, which may indicate that it can stabilize the binding pocket of 1ME3.

#### 3.5.4 Protein secondary structure elements

The comparative analysis of secondary structure elements (SSE) in the 1ME3 protein highlights notable differences in structural organization and stability over a 200 ns molecular dynamics (MD) simulation. The SSE histogram plots reveal that 1ME3 consistently exhibits prominent α-helices (in red) and β-strands (in blue) across its residue indices. This continuous and broader distribution of secondary structures reflects a well-organized and stable protein conformation. As shown in Figure 9, 1ME3 maintains an overall SSE content of 40.54%, comprising 19.45% α-helices and 21.09% β-strands. These values indicate a slightly more ordered and stable secondary structure. Furthermore, the SSE timeline plots (Figure 9) confirm that 1ME3 preserves its secondary structure throughout the simulation, with minimal structural deviations. Overall, the data suggest that the 1ME3 protein retains its structural integrity and demonstrates marginally enhanced conformational stability, as evidenced by higher SSE content and reduced fluctuations during the MD simulation.

#### 3.5.5 Principal component analysis (PCA)

**FIGURE 10:**
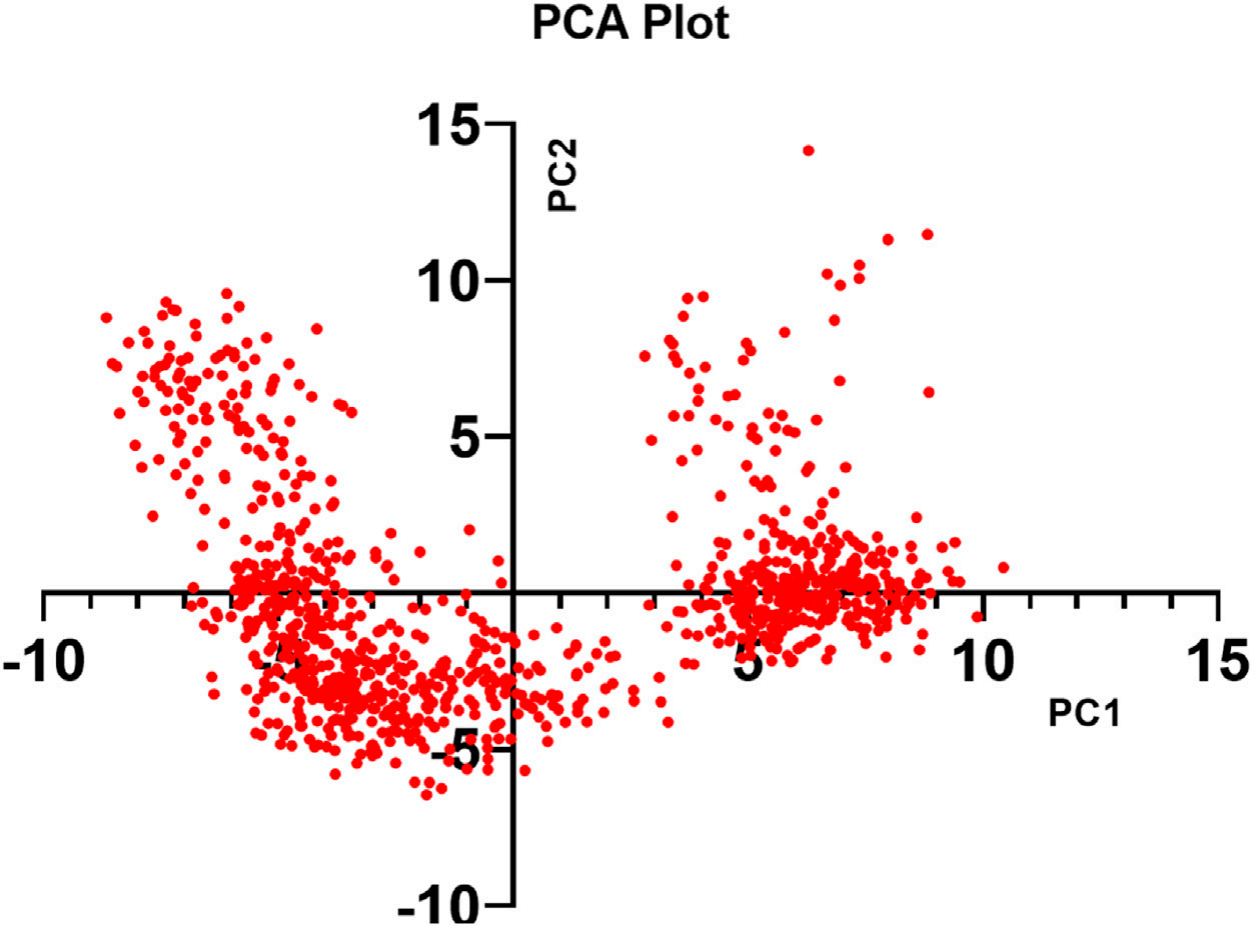
PCA of F6609-0134-1ME3 protein-ligand complex.

Throughout the simulation, the PCA method was used to examine the protein’s conformational distribution and large-scale collective motions within the protein-ligand complex. Using the Desmond script (trj_essential_dynamics.py), Essential Dynamics (ED) analysis calculated the primary components of Cα atoms to anticipate the dynamic behavior of the protein. Except for PC1 and PC2 negative modes, phase-space projection along PC1 showed a consistent conformational distribution. RMSD, RMSF, and PCA values derived from MD simulation trajectories verified the stability of the F6609–0134–1ME3 complex in dynamic states (Figure 10).

#### 3.5.6 Molecular mechanics/generalized born surface area (MM-GBSA)

The free binding energy of the ideal molecule, F6609-0134, which exhibited the highest docking score and predicted activity, was analyzed based on its molecular dynamics (MD) simulation frames. Over a 0–200 ns MD trajectory, the total average binding energies were calculated as follows: ΔG Bind (−39.84 kcal/mol), ΔG Bind H-bond (−0.67 kcal/mol), ΔG Bind Lipo (−14.81 kcal/mol), and ΔG Bind vdW (−38.96 kcal/mol). Analysis of these values, as presented in Table 6, indicates that ΔG Bind and ΔG Bind vdW contributed most significantly to the overall binding energy, emphasizing the role of van der Waals interactions in molecular stability.

**TABLE 6:** Free binding energies of the molecule F6609-0134 shown through MM-GBSA.*

MD snapshot (ns)	ΔG bind	ΔG bind H-bond	ΔG bind lipo	ΔG bind vdW
0	−42.53	−0.44	−19.28	−41.31
20	−44.91	−0.32	−12.51	−44.77
40	−48.29	−0.65	−11.28	−43.58
60	−36.16	−0.09	−14.18	−43.65
80	−43.75	−0.28	−16.17	−44.75
100	−31.46	−1.30	−12.02	−27.83
120	−38.84	−1.05	−14.46	−34.12
140	−40.92	−0.10	−19.65	−41.10
160	−42.44	−0.86	−17.16	−39.31
180	−35.11	−1.33	−13.02	−37.02
200	−33.87	−1.00	−13.20	−31.12
Average	**−39.84**	**−0.67**	**−14.81**	**−38.96**

*kcal/mol.

Stable van der Waals contacts with important amino acid residues were found by analyzing the ΔG Bind vdW values for F6609-0134 interactions with the protein complex. It was discovered that the binding energies derived from MM-GBSA computations based on MD simulation trajectories and docking investigations were consistent. Interestingly, the molecule’s low free binding energy suggested that it had a high affinity for the receptor. These results support F6609-0134’s potential as a promising inhibitor by indicating that it interacts strongly with 1ME3.

## 4 Conclusion

Trypanosoma cruzi causes Chagas disease, a neglected tropical illness that continues to pose a serious threat to world health because of the lack of effective treatments and medication resistance. The potential of machine learning-driven QSAR modeling to speed up the drug discovery process for Chagas disease is demonstrated in this work. The model exhibiting the highest predictive accuracy among those assessed was the ANN-based QSAR model utilizing CDK fingerprints. The stability and high binding affinity of F6609-0134 were confirmed using molecular docking studies and molecular dynamics simulations, which further substantiated its selection as a possible lead molecule. According to these findings, F6609-0134 is a promising therapeutic option for *Trypanosoma cruzi* that needs more experimental support. The molecule should be manufactured or purchased commercially to enhance its potential as an anti-Chagas agent, and thorough *in vitro* tests aimed at *T. cruzi* should be used to evaluate its effectiveness. In the end, these follow-up investigations will promote its development as a treatment candidate for Chagas disease by confirming its trypanocidal efficacy and offering crucial information on cytotoxicity, selectivity, and mechanism of action.

## Data Availability

The datasets used in this study were curated from ChEMBL (https://www.ebi.ac.uk/chembl/), which is an open-source data repository. The code for generating the ML-assisted QSAR model can be found at: https://github.com/RatulChemoinformatics/QSAR-Models.
